# Protective and therapeutic effect of hydrogen sulfide on hemorrhagic cystitis and testis dysfunction induced with cyclophosphamide

**DOI:** 10.3906/sag-2003-10

**Published:** 2021-06-28

**Authors:** Fikriye Yasemin ÖZATİK, Orhan ÖZATİK, Yasemin TEKŞEN, Semra YİĞİTASLAN, Neziha Senem ARI

**Affiliations:** 1 Department of Pharmacology, Faculty of Medicine, Kütahya Health Sciences University, Kütahya Turkey; 2 Department of Histology and Embriology, Faculty of Medicine, Kütahya Health Sciences University, Kütahya Turkey; 3 Department of Pharmacology, Faculty of Medicine, Eskişehir Osmangazi University, Eskişehir Turkey; 4 Department of Histology and Embriology, Evliya Celebi Training and Research Hospital, Kütahya Health Sciences University, Kütahya Turkey

**Keywords:** Cyclophosphamide, hydrogen sulfide, testis, bladder

## Abstract

**Backround/aim:**

Cyclophosphamide (CP) is a drug used for treatment of many malignant diseases. However, it can cause serious side effects such as hemorrhagic cystitis and male infertility. Hydrogen sulfide (H2S) is a gaseous mediator and is suggested to have antioxidant, antiinflammatory, and antiapoptotic effects. In this study, dose-dependent effects of H2S donor sodium hydrosulfide (NaHS) on cyclophosphamide-induced hemorrhagic cystitis and testicular dysfunction were investigated in rats.

**Material and methods:**

Rats were divided into 5 groups (n = 8): control, CP, NaHS25 μmol/kg, NaHS50 μmol/kg, and NaHS100 μmol/ kg. After treatment for 7 days intraperitoneally (ip), a single ip dose of CP 200 mg/kg was given on the 8th day. Then, treatment was continued for 7 days. In bladder and testicular tissues, IL 6, IL 10, cGMP, NO, H2S, FSH, LH, and testosterone levels were measured by ELISA. Histopathological examination with H&E staining, as well as immunohistochemical staining for acrolein in bladder and caspase-3 and APAF-1 in testis were performed.

**Results:**

NaHS prevented the increased IL 6 and IL 10 values induced by CP as well as prevented the decrease in cGMP values associated with CP. There was no significant change in FSH values, but the LH value, which increased with CP, decreased with 25, 50, and 100 μmol/kg NaHS. In contrast, testosterone decreased in the CP group and increased in the treatment groups. NaHS was effective in many biochemical and histopathological parameters at 25 and 50 μmol/kg doses, and this effect decreased at 100 μmol/kg dose.

**Conclusion:**

H2S has a protective and therapeutic effect on hemorrhagic cystitis and testicular dysfunction induced by cyclophosphamide. It can be suggested that H2S is a promising molecule in facilitating cancer treatment.

## 1. Introduction 

Cyclophosphamide (CP) is currently still an antineoplastic used for treatment of many malignant diseases [1]. However, in addition to the necessity for its use, it causes serious and uncomfortable side effects in patient. Hemorrhagic cystitis and testis injury in males may be listed among these side effects [2]. Hemorrhagic cystitis is a painful disease causing many problems for the patient and unfortunately it is frequently observed during cyclophosphamide treatment [3]. The side effects of the drug are reported to be due to the active metabolite of acrolein [3]. Acrolein is excreted in urine [4,5]. As a result, the organ with most exposure to this metabolite is the bladder. During treatment with cyclophosphamide, epithelial edema, ulceration, hemorrhage and leukocyte infiltration are commonly observed in the bladder [4–6]. At the same time, there are studies stating that cyclophosphamide negatively affects fertility [7,8]. Many male cancer patients apply for sperm freezing procedures before beginning treatment to maintain reproductive capability. This is a sad and troubling situation for these people. This effect, observed as a side effect of CP in humans, is the same in experimental animals. As a result, many studies have used CP as a model with the aim of inducing hemorrhagic cystitis in experimental animals. Hydrogen sulfide (H2S) is a mediator with gas structure and is produced by mammalian tissue. H2S is synthesized from L-cysteine by two pyridoxal 5-phosphate linked enzymes, in other words cystathionine β synthase (CBS) and cystathionine γ lyase (CSE), and an enzyme independent of phosphate 3-mercaptopurine sulfur transferase (3-MST). H2S is reported to affect processes like apoptosis, inflammation, angiogenesis, proliferation, metabolism, oxygen sensitivity, and oxidative stress [9,10]. H2S is thought to play an important role in formation of visceral pain. However, antinociceptive effects have been identified. This is thought to occur through endogenous H2S upregulation by CSE via activation of the Ca (v) 3.2 channel, one of the Ca (+2) channels [11]. The analgesic effect is thought to be linked to opening of the K (ATP) channels [12]. Many studies have shown that H2S prevents inflammation and oxidative stress in ischemia/reperfusion injury and may have therapeutic effect in many organs and metabolic diseases. Additionally, there are studies which found H2S relaxed the corpus cavernosum in rabbits and increased intracavernosal pressure in animals [13,14]. Additionally, it was shown to induce the same effects in the human corpus cavernosum [15]. These effects are thought to occur through L-cysteine amino acid. In fact, in a study by La Fuante et al. H2S relaxed penile arterial and cavernous tissues in males with erectile dysfunction and they presented evidence that these effects occurred with L-cysteine mediation. The researchers proved that H2S contributed to cGMP production [16].

CP is a promising medication for many cancer patients and considering it is frequently used for this type of patient, the lack of observation of side effects will suit patients in terms of quality of life. In fact, serious side effects may represent a reason for patients to abandon this medication or leave them struggling with side effects due to necessity. H2S may be promising in terms of protecting patients from these side effects or preventing the occurrence of side effects.

Based on all this information, this study aimed to investigate the therapeutic and protective effects of H2S against hemorrhagic cystitis and testicular injury, serious side effects of the cancer drug cyclophosphamide, in male rats.

## 2. Materials and methods

### 2.1. Experimental animals and groups

In this study, 40 male Sprague Dawley rats weighing 240–280 g were used. The rats were housed at room temperature (24 ± 2 °C) with fixed limits of 55% ± 15% relative humidity and 12 h/12 h light/dark cycle. Water and standard rat feed were administered ad libitum. All procedures related to animals were completed in accordance with national and international regulations about animal experiments. The study was completed in Dumlupınar University Experimental Animals Breeding Research and Application Center and Dumlupınar University Advanced Technologies Design, Research and Development Center.

The experimental study groups were organized as follows (n = 8):

CP: cyclophosphamide, PS: physiologic serum, NaHS: sodium hydrosulfide H2S donor.

Subjects were used with the following aims.

Group 1 (Control): 0.2 mL PS intraperitoneal (ip) for 15 days duration.

Group 2 (CP): 0.2 mL PS ip 7 days before and 7 days after + 200 mg/kg ip single dose CP on the 8th day.

Group 3 (CP + NaHS25): 25 µmol/kg NaHS ip 7 days before and 7 days after + 200 mg/kg ip single dose CP on the 8th day.

Group 4 (CP + NaHS50): 50 µmol/kg NaHS ip 7 days before and 7 days after + 200 mg/kg ip single dose CP on the 8th day.

Group 5 (CP + NaHS100): 100 µmol / kg NaHS ip 7 days before and 7 days after + 200 mg/kg ip single dose CP on the 8th day.

Control group rats were administered 0.2 mL PS. The cyclophosphamide group were administered 0.2 mL PS for 7 days, then 200 mg/kg ip single dose cyclophosphamide on the 8th day (6) and finally 0.2 mL PS ip for 7 days again to complete 15 days. The treatment groups had the same protocol administered with NaHS of 25, 50, and 100 µmol/kg doses [17]. 

Animals were weighed 24 h after treatment and anesthesia was induced with ketamine (70 mg/kg ip, Ketalar, Pfizer) and xylazine (10 mg/kg ip, Alfazyne). Intracardiac blood was taken for euthanasia of animals. Blood samples were centrifuged at 1000 g for 20 min and serum was stored at 20 °C. The bladder and both testes of animals were obtained. One testis was weighed and stored at –20 °C for dry weight measurement. The other testis was divided into 2 pieces and stored for histopathologic examination and to create biochemical homogenates. The bladders of the animals were divided into 3 pieces. The 1st piece was used for histopathologic analysis, the 2nd piece was used to prepare homogenate for biochemical testing purposes, and the 3rd piece was weighed and stored at –20 °C and used to measure dry weight later.

### 2.2. Body and organ weights

Animals were weighed before and after the experiment. Weight results were recorded. Changes in body weight were calculated as percentages (BW% = (BW2–BW1)/100). Frozen testis and bladder samples were dried in an oven at 60 °C for 12 h. Dry weights were recorded and dry/wet weight ratios were calculated.

### 2.3. Histopathologic investigations

The bladder and testis were stored in 10% neutral formalin solution. Tissues were later submerged in paraffin blocks, sections were taken and stained with hematoxylin and eosin. With the aim of showing the presence of apoptotic cells, APAF-1 and caspase 3 stains were used. Additionally, the presence of acrolein in the bladder walls was assessed. For this purpose, acrolein staining was used. The Gray criteria were used for histopathologic bladder scoring. Gray scoring is grade 0: normal epithelium, grade 1: moderate degree of submucosal edema, moderate degree hemorrhage and low amounts of ulceration, and grade 2: cell infiltration, fibrin deposition, severe hemorrhage, and countless ulceration [18]. 

### 2.4. Investigation of testis and bladder homogenates

#### 2.4.1. Preparation of testis and homogenates

Tissues taken from the bladder and testis were homogenized in 5% Triton X-100 and phosphate with 1:1 ratio buffered 1 mL mixture. The homogenized tissue was later centrifuged at 1000 g for 10 min and the supernatant was used for biochemical analyses.

#### 2.4.2. Measurement of H2S, NO (nitric oxide), cGMP, IL 6 (interleukin 6), IL 10 (interleukin 10), FSH (follicle stimulating hormone), LH (luteinizing hormone), and testosterone

H2S, NO, cGMP, IL 6, IL 10, LH, FSH, and testosterone levels were measured with the aid of commercial kits. The procedures were completed in line with the guidelines in the commercial kits.

#### 2.4.3. Protein amounts in homogenates

This was measured with a maestrogen-maestronano spectrophotometer.

### 2.5. Statistical analysis

Results in are given as mean ± SD and median (%25)–(%75). Data were analyzed in the SPSS program v: 21.0 (IBM Corp., Armonk, NY) using one-way analysis of variance test (ANOVA) (posthoc Dunnett test). Comparison of histopathologic data used the Kruskal–Wallis (posthoc Dunn method) test. Significance was accepted as p < 0.05.

## 3. Results

### 3.1. Body weight values

Body weight values is shown in Table 1. When body weight (BW) ratios are investigated, the cyclophosphamide (CP) group had severe body weight loss compared to the control group (p < 0.001). The higher body weight loss in the NaHS50 group is noteworthy. There was significant body weight loss, though at lower rates, in the NaHS100 group.

**Table 1:** Body and organ weights in rats.

	Group	Mean ± SD	Median (%25)–(%75)	p
BW%	C	0.54±0.25	0.56 (0.27)–(0.78)	<0.001
	CP	–0.30 ± 0.29	–0.39 (–0.43)–(–0.03)***	
	NaHS25	–0.11±0.22	–0.03 (–0.35)–(0.10)	
	NaHS50	–0.45±0.22	–0.38 (–0.62)–(–0.30)***	
	NaHS100	–0.21±0.16	–0.30 (–0.32)–(–0.04)***	
Relative bladder weight (mg/g)	C	0.16±0.04	0.15 (0.12)–(0.19)	0.022
	CP	0.43±0.28	0.39 (0.23)–(0.71)	
	NaHS25	0.46±0.44	0.31 (0.23)–(0.46)	
	NaHS50	0.27±0.26	0.16 (0.08)–(0.44)	
	NaHS100	0,16±0.08	0.16 (0.08)–(0.22)	
Dry/wet bladder weight (mg/g)	C	0.14±0.02	0.14 (0.13)–(0.16)	0.001
	CP	0.17±0.06	0.15 (0.11)–(0.23)	
	NaHS25	0.18±0.14	0.14 (0.13)–(0.20)	
	NaHS50	0.34±0.14	0.38 (0.18)–(0.47)***	
	NaHS100	0.29±0.12	0.25 (0.20)–(0.33)***	
Relative testis weight (mg/g)	C	4.26±0.61	4.30 (4.15)–(4.75)	0.002
	CP	6.53±0.56	6.52 (6.01)–(7.08)**	
	NaHS25	5.52±0.46	5.48 (5.11)–(6.00)**	
	NaHS50	5.04±1.67	5.87 (3.30)–(6.08)**	
	NaHS100	4.89±1.46	5.15 (3.42)–(6.25)	
Dry/wet testis weight(mg/g)	C	0.13±0.01	0.13 (0.13)–(0.14)	0.007
	CP	0.19±0.07	0.17 (0.15)–(0.24)	
	NaHS25	0.36±0.58	0.16 (0.15)–(0.17)	
	NaHS50	0.16±0.01	0.16 (0.15)–(0.18)	
	NaHS100	0.16±0.02	0.16 (0.14)–(0.17)	

Values are given as mean ± SD and median (%25)–(%75). Differences are given compared to the C group (*). ** p < 0.01; *** p < 0.001.

Relative bladder weight was calculated by dividing the wet bladder weight by the final BW. Significant variation was not identified between the groups in terms of relative bladder weight. However, relative testis weight was significantly increased in CP, NaHS25, and NaHS50 groups compared to controls (p < 0.01).

Wet/dry bladder weights were observed to be significantly increased in the groups administered 50 and 100 µmol/kg NaHS compared to controls (p < 0.001). There was no significant variation in wet/dry testis weights.

### 3.2. Biochemical analysis

#### 3.2.1. Interleukin values (IL) 

**Figure 1:**
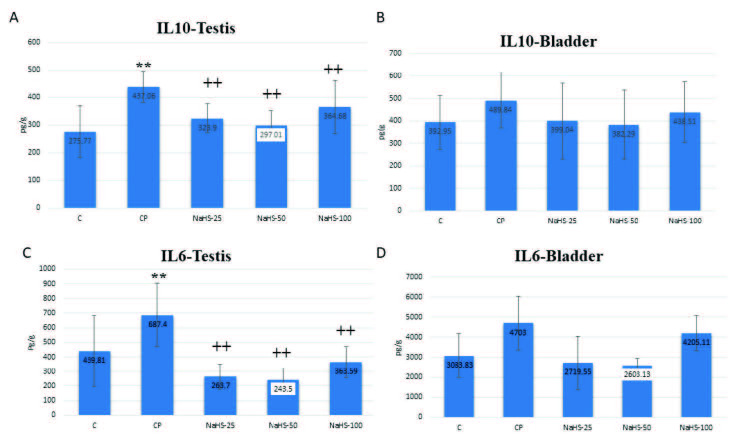
Effects of NaHS treatment on biochemical parameters in rats with cyclophosphamide toxicity. A: IL10 testis; B: IL10 bladder; C: IL 6 testis; D: IL 6 bladder. Data are given as mean   SD. (*): Different from control; ** p < 0.01; (+); Different from CP; ++ p < 0.01 ANOVA (n: 8). C: control; CP: cyclophosphamide; NaHS: sodium hydrosulfide; IL: interleukin.

Interleukin values is shown in Figure 1. When IL 10 and IL 6 values are examined, the IL 10 values increased in testis tissue in the CP group. However, the same value was lower in the groups administered 25, 50, and 100 µmol/kg NaHS compared to the CP group (p < 0.01) (Figure 1A). The greatest reduction was noted in the 50 µmol/kg NaHS group. There were no significant changes in the IL 10 values in the bladder. However, it is notable that the mean values in the 25 and 50 µmol/kg NaHS groups were closest to the control group (Figure 1B).

When examined for IL 6 values, we can see IL 6 was elevated in the CP group (p < 0.01). There was a pronounced fall in this value in the groups administered 25, 50, and 100 µmol/kg NaHS compared to the CP group. Again, when assessed in terms of mean values, the greatest reduction was observed in the 50 µmol/kg NaHS group (Figure 1C). Though there was no statistically significant variation of IL6 in bladder tissue, in terms of mean values the greatest reduction was observed in the 50 µmol/kg NaHS group (Figure 1D).

#### 3.2.2. Cyclic guanosine monophosphate (cGMP) values

cGMP values is shown in Table 2. When cGMP values are investigated, values for the bladder were identified to have significant falls in the CP, NaHS25 and NaHS50 groups compared to the control group (p < 0.01). When the NaHS100 group is compared with other treatment groups, a significant elevation is present (p < 0.01). In testis tissue, the cGMP values in the CP group were observed to be significantly low. However, this value significantly increased, especially in the group administered 50 µmol/kg NaHS. The same increase was observed in the other treatment groups, with the highest increase in mean values observed in the 50 µmol/kg NaHS group.

**Table 2:** cGMP, NO, H2S bladder and testis values.

		Group	Mean ± SD	Median (%25)–(%75)	p
cGMP	Bladder	C	315.09±79.56	304.30 (243.33)–(378.13)	0.009
		CP	195.70±19.71	196.44 (178.21)–(212.35)**	
		NaHS25	221.97±30.31	218.92 (201.67)–(246.83)**	
		NaHS50	224.78±37.6	239.34 (190.00)–(255.00)**	
		NaHS100	287.62±66.38	295.45 (215.72)–(338.75)++	
	Testis	C	23.28±16.22	15.62 (10.62)–(42.14)	0.002
		CP	5.72±2.51	5.35 (4.32)–(7.27)	
		NaHS25	18.90±10.59	15.81 (10.82)–(29.23)++	
		NaHS50	37.68±15.86	38.30 (22.51)–(52.15)++	
		NaHS100	32.83±23.56	26.53 (11.13)–(61.10)++	
NO	Bladder	C	24.73±9.48	21.88 (17.54)–(36.09)	0.05
		CP	18.70±3.89	19.09 (14.38)–(22.38)	
		NaHS25	25.85±7.16	25.23 (20.50) –(31.67)	
		NaHS50	32.15±6.24	31.59 (26.56)–(38.85)*+	
		NaHS100	26.21±9.06	25.86 (17.38)–(36.17)*+^	
	Testis	C	0.71±0.52	0.71 (0.17)–(1.13)	0.006
		CP	0.46±0.28	0.36 (0.25)–(0.68)	
		NaHS25	1.04±0.43	1.04 (0.80)–(1.44)	
		NaHS50	1.74±0.48	2.03 (1.13)–(2.07)**++	
		NaHS100	0.91±0.45	0.97 (0.42)–(1.30)^^	
H2S	Bladder	C	79.04±18.24	76.34 (61.36)-(96.81)	0.019
		CP	60.81±26.03	65.00 (42.05)-(76.67)	
		NaHS25	78.93±22.47	79.67 (58.65)-(100.68)	
		NaHS50	108.14±37.98	117.69 (77.11)-(134.13)**++	
		NaHS100	116.37±20.73	116.59 (95.15)-(138.64)**++	
	Testis	C	0.94±0.56	0.92 (0.39)–(1.44)	0.001
		CP	0.64±0.40	0.54 (0.30)–(1.03)	
		NaHS25	1.29±0.27	1.18 (1.12)–(1.48)***+++	
		NaHS50	1.99±0.35	2.12 (1.64)–(2.29)***+++	
		NaHS100	2.43±0.77	2.61 (1.87)–(3.05)***+++	

Values are given as mean ± SD and median (%25)–(%75). Differences are given compared to the C group (*), CP group (+), NaHS50 (^). * p < 0.05; ** p < 0.01; *** p < 0.001, + p < 0.05; ++ p < 0.01; +++ p < 0.001, ^ p < 0.05; ^^ p < 0.01.C: control; CP: cyclophosphamide; NaHS: sodium hydrosulfide.

#### 3.2.3. Nitric oxide (NO)-hydrogen sulfide (H2S) values

Nitric oxide (NO)-hydrogen sulfide (H2S) values is shown in Table 2. When NO and H2S values are investigated, the NO values in testis tissue appeared to be significantly elevated in the NaHS50 group compared to both the control group and the CP group (p < 0.01). The NaHS100 group had a significant fall compared to values in the NaHS50 group (p < 0.01). Similarly, in bladder tissue there was a significant elevation in the NaHS50 group (p < 0.05).

The H2S values in testis tissue were significantly elevated in all three treatment groups compared to the control and CP groups (p < 0.001). The greatest increase in mean value was observed in the NaHS100 group. When bladder tissue is examined, there were significant increases in the 50 and 100 µmol/kg NaHS groups compared to the control and CP groups (p < 0.01).

#### 3.2.4. Follicle stimulating hormone (FSH), luteinizing hormone (LH), and testosterone (T) values

**Figure 2:**
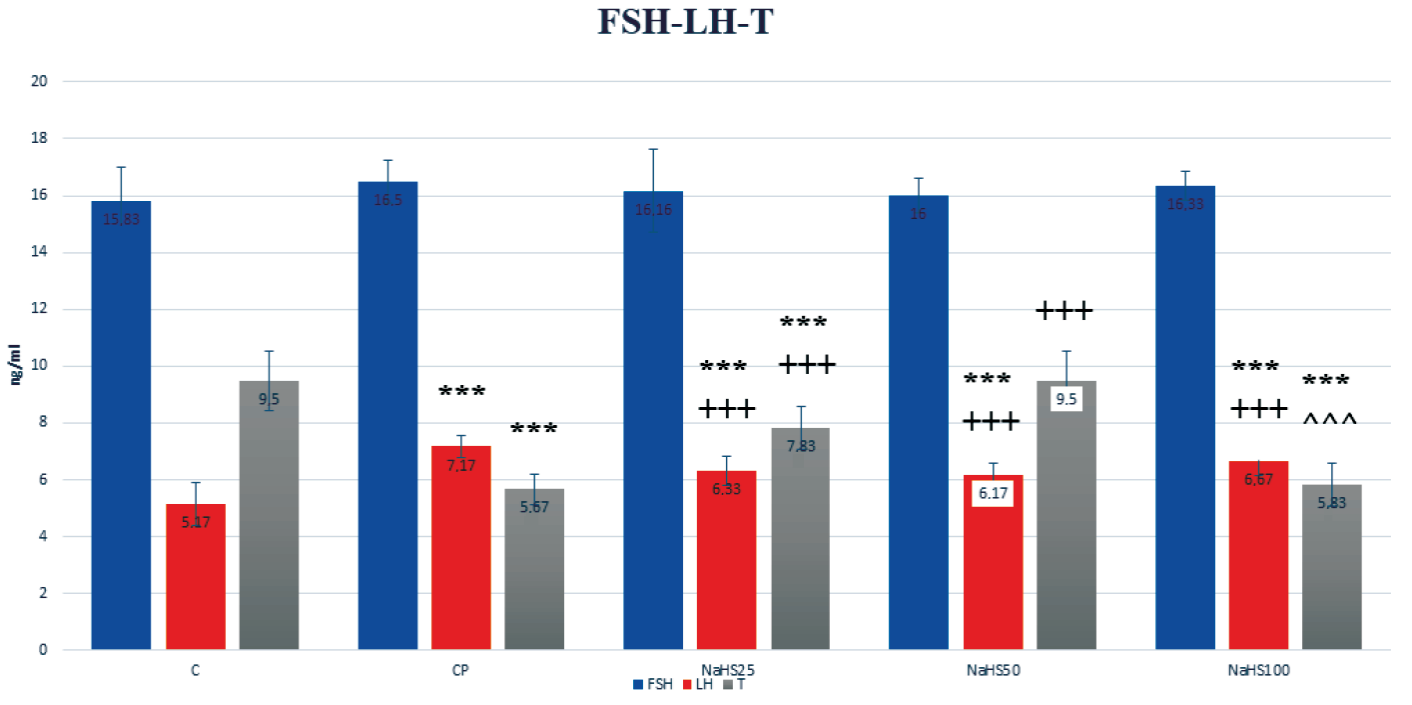
Effects of NaHS treatment on hormonal parameters in rats with cyclophosphamide toxicity. Data are given as mean   SD. (*): different from control ; *** p < 0.001; (+); different from CP; +++ p < 0.001; (^): different from NaHS50; ^^^ p < 0.001 ANOVA (n: 8). C: control; CP: cyclophosphamide; NaHS: sodium hydrosulfide; FSH: follicle stimulating hormone; LH: luteinizing hormone; T: testosterone.

Follicle stimulating hormone (FSH), luteinizing hormone (LH), and testosterone (T) values are shown in Figure 2. The FSH values had no differences observed between the groups. However, when the LH values are examined, there was a significant increase identified in the CP group compared to the control group (p < 0.001). There were significant falls identified in the other treatment groups compared to both control and CP groups (p < 0.001). When testosterone levels are investigated, there was a significant reduction observed in the CP group (p < 0.001). When the values in the 25 and 50 µmol/kg NaHS groups are investigated, there was a increase in testosterone values compared to the CP group (p < 0.001). The value in the 100 µmol/kg NaHS group was significantly low compared to the NaHS50 group (p < 0.001). However, testosterone levels were only identified to reach control levels in the NaHS50 group. This group was not identified to have any significance compared to control.

#### 3.2.5. Histopathologic values

##### 3.2.5.1. Bladder hematoxylin & eosin (H&E) staining

**Figure 3:**
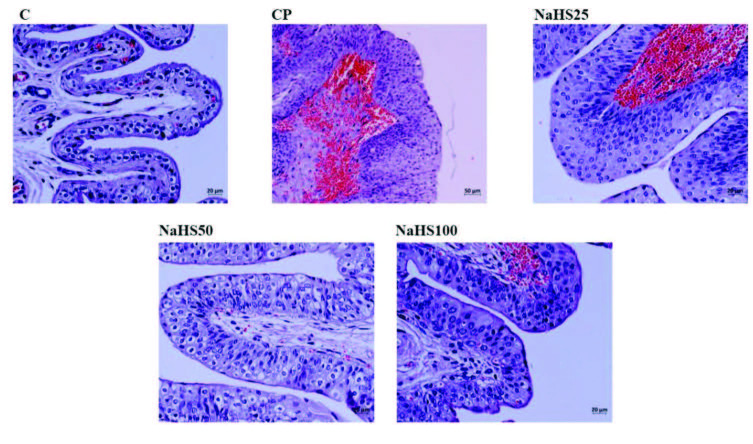
Representative images of H&E staining in the bladder. C: control; CP: cyclophosphamide; NaHS: sodium hydrosulfide.

Bladder hematoxylin & eosin (H&E) staining is shown in Figure 3. Bladder preparates stained with H & E had normal bladder epithelium appearance in the control group. In the CP group, bladder mucosa epithelium had mild degree of desquamation, severe hemorrhage in lamina propria and submucosa, and mild levels of inflammatory cell inflammation observed (grade 2). In the NaHS25 group, the bladder epithelium had partially normal appearance with severe hemorrhage in the lamina propria and submucosa and mild levels of inflammatory cell infiltration similar to the CP group. The NaHS50 group had bladder epithelium with appearance close to the control group for lamina propria and submucosa (grade 0). The NaHS100 group had less desquamation of bladder epithelium compared to the CP group, and milder hemorrhage in lamina propria and submucosa and inflammatory cell infiltration compared to the NaHS25 group (grade 1). 

##### 3.2.5.2. Bladder immunohistochemical staining

**Figure 4:**
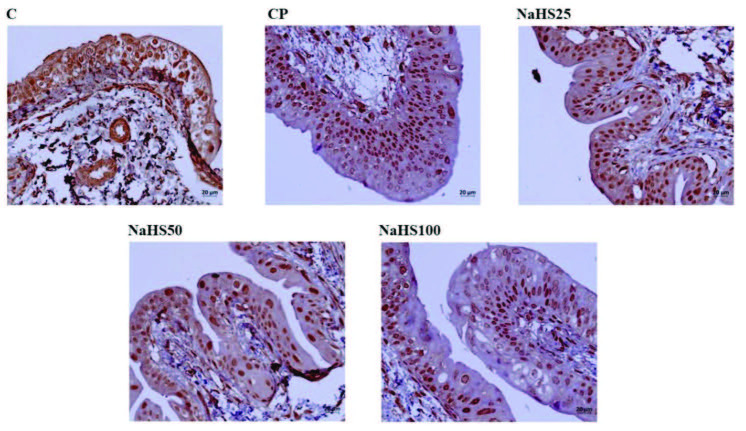
Representative images of APAF-1 staining in the bladder. C: control; CP: cyclophosphamide; NaHS: sodium hydrosulfide.

**Figure 5:**
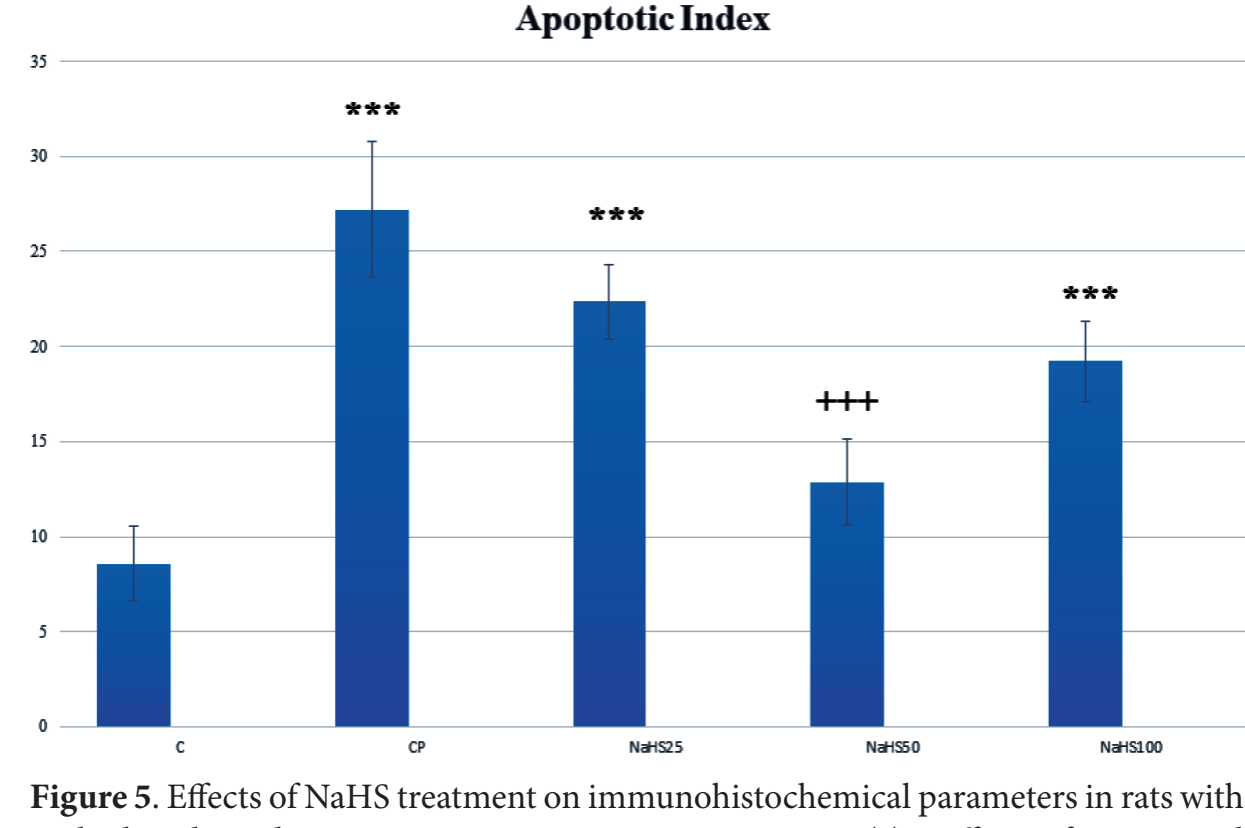
Effects of NaHS treatment on immunohistochemical parameters in rats with cyclophosphamide toxicity. Data are given as mean   SD. (*): Different from control ; *** p < 0.001; (+); Different from CP; +++ p < 0.001 ANOVA (n: 8). C: control; CP: cyclophosphamide; NaHS: sodium hydrosulfide.

**Figure 6:**
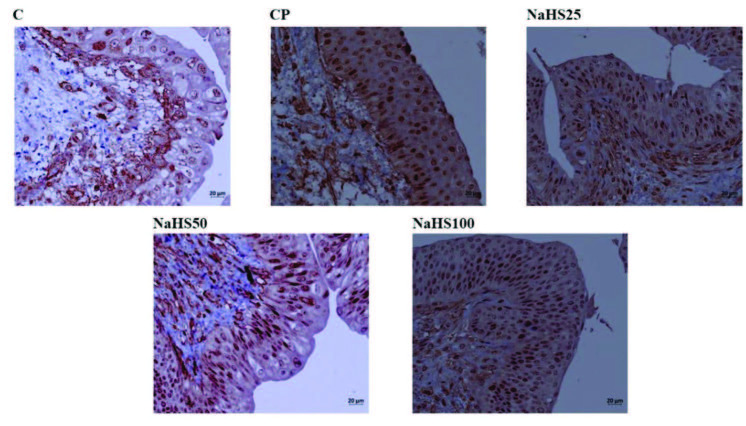
Representative images of Caspase-3 staining in the bladder. C: control; CP: cyclophosphamide; NaHS: sodium hydrosulfide.

**Figure 7:**
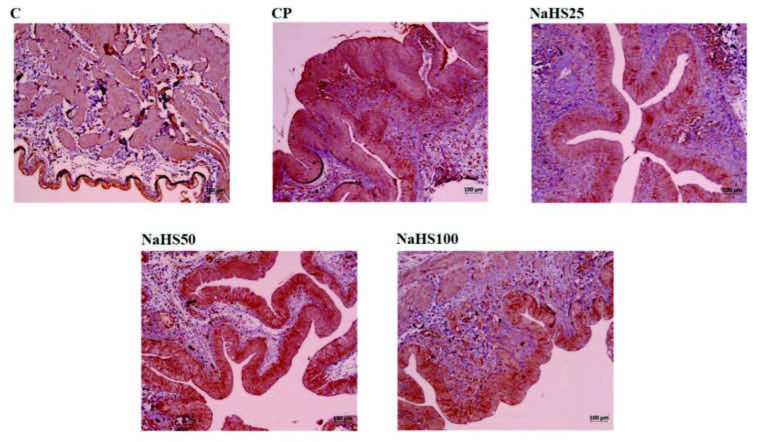
Representative images of Acrolein staining in the bladder. C: control; CP: cyclophosphamide; NaHS: sodium hydrosulfide.

Bladder immunohistochemical staining is shown in Figure 4, Figure 5, Figure 6, and Figure 7. Apoptotic cell counts with immunohistochemical staining observed an increase in apoptotic cell numbers especially in the CP group (Figure 4, Figure 5, and Figure 6). Additionally, a notable finding is that the NaHS50 group had a significant reduction in apoptotic cell counts compared to the CP group (Figure 4, Figure 5, and Figure 6).

Acrolein staining did not observe any areas stained for acrolein in the control group bladder (Figure 7). However, very darkly stained areas were present in the CP group (Figure 7). In the treatment groups, the group with least acrolein staining was observed to be the NaHS50 group (Figure 7). 

##### 3.2.5.3. Testis H&E staining

**Figure 8:**
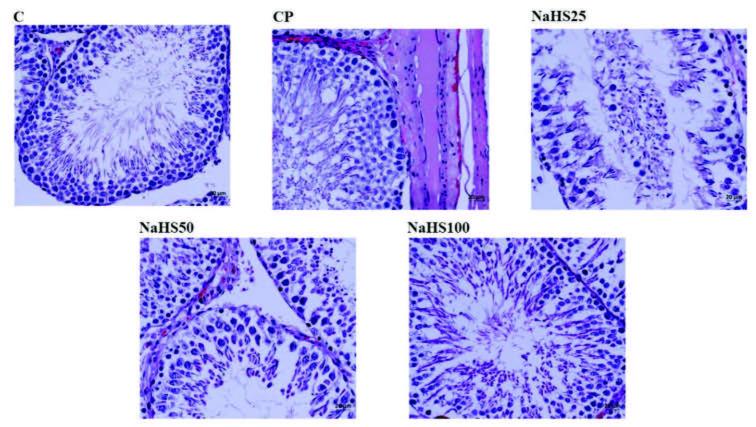
Representative images of H&E staining in the testis. C: control; CP: cyclophosphamide; NaHS: sodium hydrosulfide.

**Figure 9:**
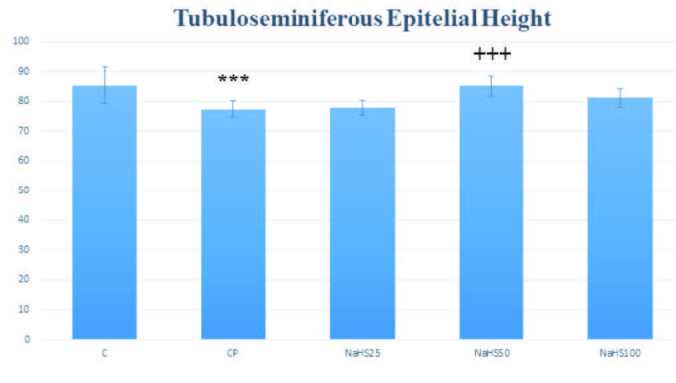
Effects of NaHS treatment on histological parameters in rats with cyclophosphamide toxicity. Data are given as mean   SD. (*): Different from control; *** p < 0.001; (+); Different from CP; +++ p < 0.001 ANOVA (n: 8). C: control; CP: cyclophosphamide; NaHS: sodium hydrosulfide.

Testis H&E staining is shown in Figure 8, Figure 9. Histological investigation of testis tissue found testis sections from the control group contained seminiferous tubules, cells belonging to spermatogenic series and Leydig cells in interstitial tissue had normal structure and no pathologic findings were encountered (Figure 8). Testis sections from the CP group observed intense hemorrhage and edema in the interstitial area, increased seminiferous tubule numbers with germinal cells falling into the lumen, occasional vacuolization in germinal epithelium, and reduced thickness of germinal epithelium (Figure 8). Testis sections from the NaHS25 group observed an improvement in hemorrhage and edema in the interstitial area seen in the CP group, with similar increases in seminiferous tubules falling from germinal cells into the lumen and reductions in germinal epithelium thickness (Figure 8). Sections from the NaHS50 group had occasional hemorrhage and edema in the interstitial area, with germ cell counts falling into the lumen in seminiferous tubules and germinal epithelium thickness similar to the control group (Figure 8). The NaHS100 group had hemorrhage and edema in the interstitial area rarely but occasionally, and vacuolization in germinal epithelium, with germ cell numbers falling into the lumen in seminiferous tubules and germinal epithelium thickness similar to the control group (Figure 8).

Tubuloseminiferous epitelyal height was reduced in the CP group compared to the control group (p < 0.001). In the NaHS50 group, there was a significant increase observed compared to the CP group (p < 0.001) (Figure 9). 

##### 3.2.5.4. Testis immunohistochemical staining

**Figure 10:**
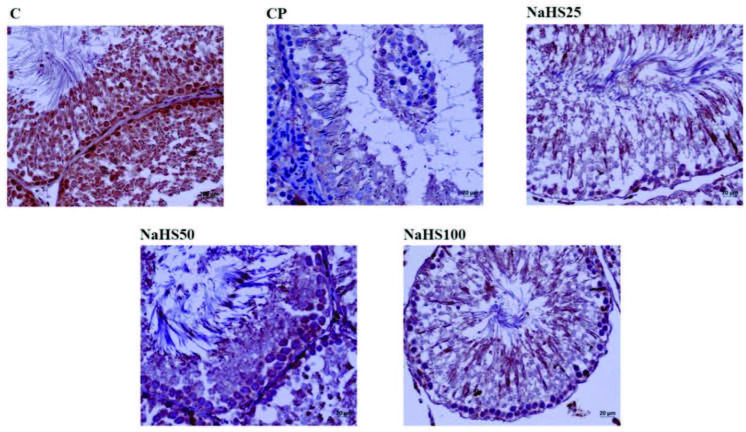
Representative images of Acrolein staining in the testis. C: control; CP: cyclophosphamide; NaHS: sodium hydrosulfide.

Testis immunohistochemical staining is shown in Figure 10. Immunohistochemical staining observed increased apoptotic cell counts especially in the CP group. The NaHS50 group was observed to have a significant reduction in apoptotic cell counts compared to the CP group.

## 4. Discussion

In this study we aimed to investigate the therapeutic and protective effects of H2S against hemorrhagic cystitis and testicular injury, serious side effects of the anticancer cyclophosphamide, in male rats. So, we investigated IL 6, IL 10, cGMP, NO, H2S, FSH, LH, and testosterone values.

We identified a significant fall in IL 6 and IL 10 values, especially in the testes of treatment groups administered 25 and 50 µmol/kg NaHS. However, a significant increase was present in the group administered CP. Stimulation of the bladder with CP causes excessive inflammation. As a result, the increase in IL 6 levels in the CP group is accepted as normal. In fact, a study identified that CP increased IL 6 levels [19]. IL 6 expression is linked to protein kinase signaling activated by p38-mitogen and c-jun pathway activated by NF k B. H2S is known to have a regulatory role in inflammation. A study of cattle mammary infections identified exogenously-administered H2S lowered IL 6 levels [20]. Similar results were obtained in our study. In the testes, pronounced falls in IL 10 and IL 6 levels were observed, especially with 50 µmol/kg NaHS dose. Interestingly, these levels were lower than the levels with dose of 100 µmol/kg NaHS. The IL 6 and IL 10 levels increased with administration of 100 µmol/kg NaHS dose. We observed the same effects in our study as in our previous study where we researched the effective dose of exogenous H2S [21].

Used as a secondary messenger in signal transduction pathways, cGMP regulates a variety of physiologic functions. Smooth muscle contractility, neurotransmission and cell growth and proliferation may be listed among these functions. In our study, we observed cGMP levels fell in both bladder and testis tissue with administration of CP. In fact, we observed similar results in our previous study. A study by De Oliviera et al. [22] induced cystitis in mice with CP and investigated the effects of an agent named BAY 58-2668 on cystitis. The researchers observed that CP caused a fall in cGMP levels. However, the agent called BAY 58-2667 did not ameliorate this effect and as a result did not improve bladder functions. This study was similar to our study. There was no study encountered investigating the effects of H2S. We observed that cGMP levels fell in the group administered CP and that the cGMP levels increased in the groups administered NaHS. In testis tissue the 50 µmol/kg NaHS dose was effective especially, while in bladder tissue all three doses were effective; however, we observed the most effective dose was 100 µmol/kg NaHS.

When we examined NO and H2S values, we observed NO values were increased in both bladder and testis of the group administered 50 µmol/kg NaHS. The formation of the guanyl cyclase enzyme, which converts GTP to c-GMP, is accelerated by NO, carbon monoxide (CO) and natriuretic peptide (NUP). A small molecule, NO is formed with the help of nitric oxide synthase (NOS) enzyme in the presence of L-arginine and oxygen [23]. NO released from urothelium or nerve fibers affects efferent and afferent neurotransmission. This causes inhibitory effects in bladder smooth muscle. NO nucleoside has the effect of increasing catalytic activity of enzyme-soluble guanyl cyclase (sGC), a cytosolic, heterodimeric protein comprising α - and β-subunits and a protetic heme group including a β-subunit transforming GTP to cGMP [22]. Once formed, NO enters the smooth muscle cell, it increases the level of cGMP over the soluble guanyl cyclase enzyme, the second increased messenger molecule (cAMP and cGMP) within the cytoplasm stimulates the protein kinase enzyme in the cell and finally, Ca level decreases in cytoplasm and smooth muscle relaxation is provided [23]. In other words, cGMP and NO are linked to each other. Studies have shown a common signal path mediating the effects of NO and H2S on vascular functions like vasodilatation, vascular remodeling (migration and proliferation), and angiogenesis. Recent studies have shown H2S-mediated NO regulation has a role in regulating angiogenesis and reducing ischemia-reperfusion injury. Kolluru et al. [24] explained the protective effects of H2S and donors against proangiogenic and ischemia/reperfusion injury may occur via induction of effectors in the VEGF/VEGFR2 signals and vascular endothelial cells PI3K/Akt/eNOS. Additionally, H2S prevents degradation of eNOS and later is reported to induce NO production by eNOS phosphorylation via PI3K/Akt activity and p38 MAPK pathway mediation. The pharmacologic donors of NO upregulate substrate bioavailability and expression for the H2S synthesis enzyme CSE and may result in H2S production occurring with vasodilator effects [24]. This information complies with the results of our study. 

We observed H2S increased in a dose-dependent manner in testis tissue. However, when we examined bladder tissue, the highest level of H2S was observed in the group administered 50 µmol/kg NaHS. This result seems that this dose was effective for hemorrhagic cystitis. H2S is an effective gasotransmitter in both bladder and testis. Both CBS and CSE are expressed in the human corpus cavernosum. Exogenously-administered H2S was observed to relax isolated corpus cavernosum endothelium independently. The testis is a structure sensitive to oxidative stress. A study by Wang et al. [25] identified a reduction in H2S concentrations in seminal plasma of infertile patients. Administration of H2S externally was identified to resolve this problem. Finally, they concluded that exogenous H2S protected testicular function and was effective in terms of fertility.

H2S is predicted to be effective in the bladder. Cystathionine synthase is expressed in the human bladder and produces H2S at identifiable rates in vitro. Additionally, H2S is a mediator of the smooth muscle relaxant effect of phosphodiesterase 5 (PDE-5) [26]. At the same time, H2S may be produced endogenously in rat and mouse bladders. Animal studies have shown that H2S is effective on bladder smooth muscle relaxation. This effect of H2S occurs by activation of the potassium-ATP dependent channel in the human bladder. Both CSE and CBS enzyme are expressed in human urothelium and detrusor muscle and produce H2S. CBS regulates phosphorylation activity within urothelium. This enzyme makes phosphorylation more active dependent on cyclic guanosine monophosphate/protein kinase (cGMP/PKG) especially in Ser 227. Stated differently, when there is an increase in cGMP, there is also an increase for H2S and this relaxes the bladder [27]. These results comply with the results of our study. In our study we observed a significant increase in H2S levels in the bladder, especially in the group administered 50 µmol/kg NaHS. Additionally, when compared with the CP groups, we observed significant increases in cGMP levels. According to these results, the administered NaHS as H2S donor can be said to have protective and therapeutic effect on toxicity induced by CP in the bladder and testis.

Additionally, we investigated FSH, LH and testosterone levels to assess testis functions. LH values were higher in the group administered CP; however, testosterone levels were low. This result complies with the results of previous studies. In fact, a study which induced dyszoospermia with CP obtained the same results [28]. When we look at the treatment groups, the decrease in testosterone values in CP group were found to increase. We determined that the closest rise to the control values was in the group in which we administered 50 µmol/kg NaHS. In the group administered 100 µmol/kg NaHS, we observed LH levels began to rise and testosterone levels began to fall again. According to these results, we can say H2S has a positive effect on testis dysfunction. LH is a hormone produced by the pituitary gland in the brain. In males, LH causes production of testosterone by stimulating Leydig cells in the testes. After testis dysfunction induced by CP, it is natural that testosterone production will stop or reduce. On the contrary, the pituitary gland begins to produce more LH. Considering the increase in testosterone levels with of, exogenous H2S may be concluded to be effective on cases with drug-related infertility. In fact, histologic and histopathologic results comply with these findings.

In our study, we performed histologic staining with H&E. The findings here support our biochemical results. Very intense hemorrhagic foci were observed in the CP group, while appearances close to the control group were observed in the NaHS50 group especially [29]. We used APAF-1, caspase-3, and acrolein staining for immunohistochemical studies. APAF-1 and caspase 3 staining provided results compatible with H&E staining. Here, the apoptotic cell counts were increased in the CP group; however, we identified these numbers were much reduced especially in the NaHS50 group. These results were the same for both bladder and testis. 

When all these findings are evaluated, H2S has protective and therapeutic effects on hemorrhagic cystitis and testis dysfunction developing linked to the cancer drug CP. Medications developed with this gasotransmitter will protect cancer patients from the side effects of drugs and/or treat the side effects caused by drugs with the effective dose of 50 µmol/kg. H2S is a promising molecule for the development of new medications.

## Funding

This study was supported by Dumlupınar University, Scientific Research Project Coordinator (DPUBAP–2018-12).

## Compliance with ethical standards

 The approval of this study was obtained from the Animal Experiments Local Ethics Committee of Dumlupınar University (2017.12.03).
